# Cardiac regeneration following myocardial infarction: the need for regeneration and a review of cardiac stromal cell populations used for transplantation

**DOI:** 10.1042/BST20210231

**Published:** 2022-02-07

**Authors:** Rita Alonaizan, Carolyn Carr

**Affiliations:** Department of Physiology, Anatomy and Genetics, University of Oxford, Oxford OX1 3PT, U.K.

**Keywords:** cardiac progenitor, cell therapy, heart regeneration, myocardial infarction

## Abstract

Myocardial infarction is a leading cause of death globally due to the inability of the adult human heart to regenerate after injury. Cell therapy using cardiac-derived progenitor populations emerged about two decades ago with the aim of replacing cells lost after ischaemic injury. Despite early promise from rodent studies, administration of these populations has not translated to the clinic. We will discuss the need for cardiac regeneration and review the debate surrounding how cardiac progenitor populations exert a therapeutic effect following transplantation into the heart, including their ability to form *de novo* cardiomyocytes and the release of paracrine factors. We will also discuss limitations hindering the cell therapy field, which include the challenges of performing cell-based clinical trials and the low retention of administered cells, and how future research may overcome them.

## Introduction

Myocardial infarction (MI) is a leading cause of morbidity and mortality worldwide (WHO). With advances in MI treatments, that include pharmacological agents and mechanical assist devices, more patients survive MI and therefore have an increased risk of developing heart failure. Heart transplantation, the only long-term cure available, is limited by an inadequate number of available donor hearts, the risks associated with the procedure, and the need for chronic immunosuppressants. Therefore, the search for an alternative solution is becoming increasingly more urgent.

## The need for heart regeneration

During development, cardiomyocytes arise from early cardiac progenitors or the proliferation of pre-existing cardiomyocytes [1]. Shortly after birth, cardiomyocytes undergo a final round of DNA synthesis without cytokinesis making the majority binucleated and subsequent heart growth is mainly achieved through hypertrophy [2]. Hence, the postnatal mammalian heart was once believed to be a post-mitotic organ. However, a 2009 key study provided evidence for human cardiomyocyte renewal by taking advantage of the incorporation of carbon-14, released during atomic bomb testing, into DNA [3]. It was found that cardiomyocytes renewed at a rate of 0.5 to 2%, and this rate declined with age. Multiple other studies have attempted to determine the frequency of cardiomyocyte renewal, reporting rates of ∼1% [3–5].

However, certain species possess a robust ability to regenerate their hearts, including some teleost fish and neonatal mammals. Resection of the adult zebrafish ventricle results in complete regeneration without fibrotic scar formation [6], and this is dependent on cardiomyocyte dedifferentiation and proliferation [7,8]. In contrast, Japanese Medaka fish form a fibrotic scar [9] and *Astyanax mexicanus* cave fish lost the regenerative ability in comparison with their river-dwelling counterparts [10]. The neonatal mouse retains regenerative capacity for ∼7 days following birth [11,12] which is also dependent on cardiomyocyte proliferation, suggesting this may be evolutionarily conserved. A regenerative window has also been proposed in human new-borns. Indeed, case studies demonstrated functional recovery following corrective heart surgeries in infants [13], and MI in a new-born child [14].

**Figure 1.**
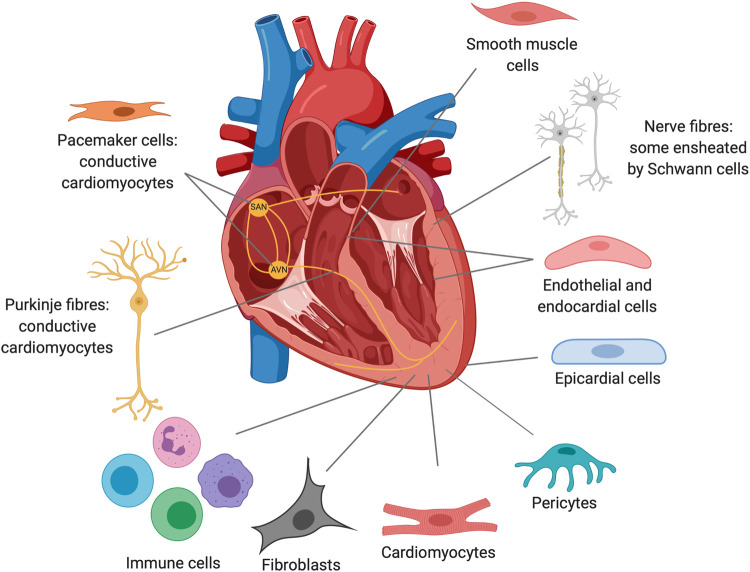
Cell types of the healthy adult heart. The main cell types of the heart are the cardiomyocytes, fibroblasts, smooth muscle cells, epicardial cells, pericytes and endothelial cells, which form the endocardium and interior lining of vasculature and valves [26]. The heart contains a network of nerve bundles and nerve fibres, some of which are encapsulated by Schwann cells [27]. Pacemaker and Purkinje cells are specialised cardiomyocytes that form the conduction system of the heart. The sinoatrial node (SAN) and the atrioventricular node (AVN) are formed by groups of pacemaker cells which generate electrical impulses and conduct them from the atria to the ventricles, respectively. Immune populations are found in the healthy adult heart including resident macrophages [28–30]. Created with BioRender.com.

Investigating these differential responses to cardiac injury provides prospects to pinpoint targets in cardiac regeneration. Several factors have been proposed to explain the differences in regenerative capacity, including polyploidisation [15], endothermy [16,17], oxygen-rich environments [18], and the immune response [19,20]. Communication between the different cell types composing the heart through both direct physical contact and paracrine signalling can also affect the reparative response (Figure 1). For example, pro-regenerative signalling from the endocardium, epicardium and macrophages has been shown to be essential for successful regeneration [19–22]. A role for cardiac neurons in the regulation of cardiomyocyte renewal has also been described. Inhibition of cholinergic transmission reduced cardiomyocyte proliferation and blunted the inflammatory response, possibly through modulation of Nrg1 and Ngf [23]. Sympathetic activity also regulates cardiomyocyte cell cycle progression through an interaction with clock genes [24]. Furthermore, cardiac damage after MI is not limited to the myocardium, but also includes other components such as the cardiac vasculature. Therefore, targeting these components may be needed for true cardiac repair [25].

## Stromal cell types for cardiac transplantation

As discussed, the main contribution to cardiomyocyte renewal in the adult mammalian heart is cardiomyocyte proliferation but this occurs at a very low level. However, several cell populations including stromal cells, such as mesenchymal stem cells, and cardiac progenitor cells (CPCs) have been investigated for transplantation with the aim of replacing lost cells following MI. Others have investigated the delivery of terminally differentiated cardiomyocytes or cardiac-committed cells expressing developmental progenitor markers derived from pluripotent stem cells (PSCs). This review will focus on stromal cells, which will be defined and discussed in the following sections.

### Mesenchymal stem cells

The term ‘mesenchymal stem cells’ (MSCs) was first coined in 1991 by US biologist Caplan to describe a population of cells isolated from bone marrow [31]. The cells were shown to adhere to plastic and differentiate into osteocytes, chondrocytes, and adipocytes. Since then, cells with similar characteristics have been described in cultures of other foetal and adult tissue including the heart [32]. The origin of tissue-resident MSCs is debated but it has been suggested that most are derived from the perivascular cells, pericytes. Pericytes isolated from a variety of tissues give rise to MSCs as identified by their cell surface markers and *in vitro* multipotency, although this plasticity may be the result of the artificial cell culture environment *in vitro* [33]. MSCs have also been reported to give rise to endothelial cells [34], cardiomyocytes [35], and insulin-producing cells [36], among others. MSCs have been claimed to be hypoimmunogenic [37] and safe for allogeneic transplantation, though this was later challenged [38]. This made MSCs an attractive tool for allogenic cell therapy and research on MSCs has been on the rise with more than 55 000 articles published so far [39].

A range of different markers are used to characterise MSCs. In 2014, a study showed that proposals submitted to the US FDA for MSC-based products did not agree on the tissue sources, manufacturing processes, and molecular characterisation of MSCs [40]. Moreover, it has been shown that MSCs isolated from different tissue varied considerably in their gene expression profile and differentiation potential. This emphasised the need to discern standardised criteria which define an MSC, although attempts to do this by the International Society for Cellular Therapy in 2006 had limited success [41].

Moreover, there is controversy about the stemness and functional mechanisms of MSCs due to the lack of evidence of direct regeneration following transplantation. Indeed, Caplan called for a name change from ‘mesenchymal stem cells’ to ‘medicinal signalling cells’ to highlight their paracrine mechanism of action rather than direct contribution to tissue [39,42]. The pro-angiogenic and anti-apoptotic properties of the MSC secretome have been demonstrated in many studies [43–45]. For example, MSC-derived exosomes induced cardiomyocyte autophagy via AMPK and Akt pathways and miRNA-regulated cell survival pathways [45]. MSC transplantation accelerated angiogenesis and improved heart function following cardiac damage in large animal models such as swine [46] and nonhuman primates [44]. MSCs have been shown to be safe and demonstrated conflicting results in clinical trials [47] and there are currently over 30 ongoing clinical trials of MSC transplantation for cardiac repair. Nonetheless, there is a need for large-scale clinical trials to support the efficacy of this approach as an allogenic off-the-shelf treatment.

### Proposed populations of adult mammalian cardiac progenitors

The suggestion that *de novo* cardiomyocytes arise from endogenous CPCs in the adult mammalian heart was, and is, controversial [4]. Nevertheless, multiple CPC isolation methods have been described that depend on the expression of proteins such as stem cell antigen 1 (SCA1), stem cell growth factor receptor KIT (KIT), Wilms tumour 1 (WT1) or islet1 (ISL1), or the ability to form colonies, form 3D multicellular clusters called cardiospheres, or to actively efflux dyes. These various populations, which are discussed below, all require expansion *in vitro* to generate sufficient cells for therapy. The resulting populations are all adherent to plastic and express some of the markers used to characterise MSCs (Table 1).

**Table 1:** Summary of markers expressed by the proposed CPC populations and MSCs

	Positive	Negative	References
*SCA1 CPCs*	SCA1, KIT^low^, CD105, CD90, CD73, GATA4, MEF2C	CD34, CD45	[48]
*KIT CPCs*	KIT, SCA1, CD105, CD166, GATA4, MEF2C	CD34, CD45	[49]
*ISL1 CPCs*	ISL1, GATA4, NKX2–5	KIT, SCA1, CD31	[49,50]
*CDCs*	SCA1, KIT^low^, CD105, CD90, CD31^low^, CD34^low^	CD45, CD133	[51,52]
*Epicardium-derived cells*	KIT^low^, CD105, CD90, CD73, CD44, GATA4	CD34, CD45	[53,54]
*MSCs*	CD105, CD90, CD73	CD34, CD45, CD14, CD19, HLA-DR	[41]

#### The cardiac side population and SCA1^+^ cells

The first attempt to describe an endogenous myocardial stem cell was made by Hierlihy et al. [55] who isolated a side population (SP) from mouse hearts based on their ability to efflux the DNA-binding dye Hoechst through an ATP-binding cassette transporter. The SP represented ∼1% of the total cells in the adult heart and were described as negative for the proteins KIT, SCA1, and CD90. However, it was later shown that a CD31^−^ SCA1^+^ subset of mouse SP cells exhibited functional cardiomyogenic differentiation capacity [56] and that SP cells significantly overlapped with the SCA1^+^ population [57]. Noseda et al. [48] revealed that selected PDGFRα^+^ SP^+^ CD31^−^ cells formed the clonogenic cardiogenic subset of SCA1^+^ cells and transplantation of the clonal progeny of a single SCA1^+^ PDGFRα^+^ SP^+^ CD31^−^ cell showed cardiomyocyte, endothelial and smooth muscle lineage potential, and enhanced cardiac function, albeit with low cell retention.

Fate-mapping studies disputed the endogenous contribution of SCA1^+^ cells to cardiomyocytes in homeostasis and after MI [58,59], with cardiac SCA1^+^ cells representing a subset of vascular endothelial cells and having minimal cardiomyogenic potential. The Noseda group suggested renaming this subpopulation as cardiac mesenchymal stromal cells (cMSCs) [60] and showed that the secretome of these cMSCs suppressed cardiomyocyte apoptosis and preserved mitochondrial transmembrane potential following menadione treatment. In a mouse MI model, injecting cMSC-conditioned media reduced TUNEL+ cardiomyocytes >70% in the infarct border zone. Although an ortholog of SCA1 is absent from humans, SCA1-like cells have been isolated from the adult human heart using the mouse antibody [61]. However, SCA1 populations have not entered clinical trials.

#### KIT

In 2003, the Anversa group described a resident KIT^+^ progenitor population [62] as self-renewing, clonogenic, and negative for haematopoietic lineage markers (Lin^−^). *Kit* expression has also been described in postnatal cardiomyocytes [63], adult cardiomyocytes [64], coronary endothelial cells [65], and heart-resident cells co-expressing *CD45* indicating a bone marrow origin [66] and work from the Anversa group has since been retracted. However, in 2013 Ellison et al. [67] demonstrated the *in vivo* activation of KIT^+^ cells in the isoproterenol (ISO)-treated heart. Ablation of dividing KIT^+^ cells (and other dividing cells) abolished regeneration and functional recovery after ISO.

The phase I SCIPIO (NCT00474461) clinical trial into the delivery of autologous KIT^+^ cells has been retracted [68], but a follow-up phase II CONCERT-HF (NCT02501811) clinical trial compared the effects of MSCs, KIT^+^ cells and a combination thereof. The proportion of patients experiencing major adverse cardiac events (HF-MACE) was significantly different across the groups but none of the HF-MACE components were individually significant [69]. Moreover, there were no significant differences between treated and placebo groups in other endpoints including left ventricular ejection fraction, left ventricular volumes, and scar size.

Multiple lineage tracing studies have addressed the cardiomyogenic potential of KIT^+^ cells in the adult heart using Cre recombinase knockin approaches. It was shown that only ∼0.03% of cardiomyocytes were of the KIT lineage [70] and KIT^+^ cells were consistent with an endothelial phenotype as suggested by their co-expression of *CD31*, and their localisation within the endocardium and coronary endothelium. Following MI, only a very rare subset co-expressed the cardiogenic markers *Nkx2–5* or cardiac troponin T [64,71] and the majority of Cre-labelled cardiomyocytes were pre-existing KIT^+^ cardiomyocytes rather than cardiomyocytes formed *de novo* from KIT^+^ progenitors [64]. He et al. [72] used dual recombinase technology (Cre and Dre) to only label KIT^+^ non-cardiomyocytes and saw no contribution by these cells to *de novo* cardiomyocytes in homeostasis and after MI. The interpretation of these knockin approaches comes with caveats as they involved disruption of one KIT allele and may therefore lead to under-reporting of cells with low KIT expression [73]. Other fate-mapping studies avoided these issues by tracing all non-cardiomyocyte lineages rather than specific progenitor markers (discussed below) [74].

#### ISL1

*Isl1* expression is detected in cardiac mesodermal progenitors [75] and in cardiac neural crest cells [76]. In the developing heart, ISL1^+^ progenitors have been shown to contribute to the formation of cardiomyocytes, endothelial cells and smooth muscle cells [77]. In 2005, Laugwitz et al. [50] showed that ISL1^+^ cells give rise to a minor proportion of cardiomyocytes in the postnatal murine heart, although the distribution and numbers of ISL1^+^ cells are not altered following MI [78]. More recently, transplantation of embryonic stem cell (ESC)-derived ISL1^+^ progenitors improved cardiac function following MI demonstrating the validity of transplanting developmental cardiac progenitors with the aim of replacing lost cardiomyocytes [79]. The ESCORT clinical trial (NCT02057900) demonstrated the safety of transplanting hESC-derived ISL1^+^ cells embedded in a fibrin patch [80]. However, the study was limited by the small sample size of six patients only and the lack of a control group.

#### Cardiosphere-derived cells

In 2004, CDCs isolated from human and murine hearts were described as clonogenic, self-renewing and a heterogeneous population expressing *KIT*, and the mesenchymal markers *CD90*, *CD105* [51,81]. CDCs can be stimulated to differentiate into the cardiomyocyte lineage *in vitro* but at low maturation levels. This is enhanced using metabolic programming to resemble the metabolic switch from glycolysis to fatty acid oxidation that occurs in cardiomyocytes during cardiac development [82]. A therapeutic effect of CDCs has been demonstrated in pig, mouse, rat and rhesus monkey [51,83–87]. The CADUCEUS (NCT00893360) trial in MI patients showed a decrease in scar size, but no changes in left ventricular ejection fraction [88]. The follow-up phase II ALLSTAR (NCT01458405) clinical trial was terminated in 2019 for failing to meet the primary end point of scar size reduction, although a significant improvement in segmental myocardial function was observed in the CDC group [89,90]. In the CAREMI phase I/II trial (NCT02439398), CDC-like cells were isolated by positive immunomagnetic selection of KIT and were shown to lose KIT expression during *in vitro* expansion. The trial demonstrated the safety of allogenic transplantation but failed to establish efficacy [91].

More recently, CDCs have been examined at the single cell transcriptomics level [52] and described as mesenchymal/stromal/fibroblast-like with a small minority of endothelial-like cells. SCA1^+^ CDCs showed pro-angiogenic capabilities, whereas SCA1^−^ CDCs had higher angiogenic potential, mediated by Vegfa and Flt1 interactions. Interestingly, transplantation of SCA1^+^ CDCs but not SCA1^−^ CDCs improved cardiac function after MI. This was attributed to the secretion of cardioprotective ligands such as Cxcl12 and Hgf from SCA1^+^ CDCs, demonstrating the therapeutic benefit of the CDC secretome and the previously unappreciated functional differences between CDC subpopulations.

#### Epicardium-derived cells

During development, the epicardium secretes trophic factors required for myocardial maturation, and undergoes EMT to directly contribute precursors of coronary vascular smooth muscle cells and fibroblasts. Epicardium-derived cells (EPDCs) were thought to contribute to the endothelial and cardiomyocyte lineages, but this was later shown to be minimal [92,93]. Following development, the epicardium becomes quiescent, but upon cardiac injury epicardial activation leads to fibrosis and scar formation in the mammalian heart [94].

Smart et al. [95] showed that WT1^+^ progenitors transplanted into infarcted hearts formed *de novo* cardiomyocytes, although the contribution of EPDCs to both vasculature and cardiomyocytes was insufficient for effective myocardial regeneration. However, co-transplantation of hESC-derived epicardial cells and cardiomyocytes resulted in the epicardial cells forming persistent fibroblast grafts that stimulated graft and host vascularisation and increased the size of cardiomyocyte grafts by inducing cell proliferation and maturation [96]. This suggests that combinatorial approaches may be an encouraging therapeutic option for cardiac repair.

## Cell therapy — mechanisms of action and current limitations

Numerous studies have questioned the differences between the various cardiac stromal cell populations including mesenchymal cells, fibroblasts, pericytes, and CPCs [97–101] due to the overlap in their morphology and gene expression profile. For example, cardiac fibroblasts have been shown to express cardiogenic transcription factors which contribute to cardiac development and repair [102], suggesting that CPCs are a subset of cardiac fibroblasts. Both cardiac and tail fibroblasts share a highly similar molecular signature to that which has been previously described for MSCs [103].

The lack of direct differentiation of delivered CPCs into cardiomyocytes has shed doubt on the identity of the proposed CPC populations. In 2017, the Cardiomyocyte Regeneration Consensus Statement noted that: (1) CPCs may contribute to the formation of cardiomyocytes in adult homeostasis, albeit at very low levels and that (2) the mechanism behind cardiomyocyte renewal in the adult mammalian heart is cardiomyocyte proliferation suggesting evolutionary conservation [104]. This is supported by studies utilising different pulse-chase approaches and fate mapping experiments that identified a sub-population of cycling cardiomyocytes as the dominant source of cardiomyocyte renewal in both homeostasis and following injury [4,105,106]. More recently, a dual genetic lineage tracing strategy in which cardiomyocytes and nonmyocytes of the developing heart could be simultaneously labelled by two orthogonal recombination systems [74] showed that nonmyocytes do not give rise to cardiomyocytes, at or beyond E11.5 to E12.5, or in the neonatal heart in both homeostasis and following injury.

**Figure 2.**
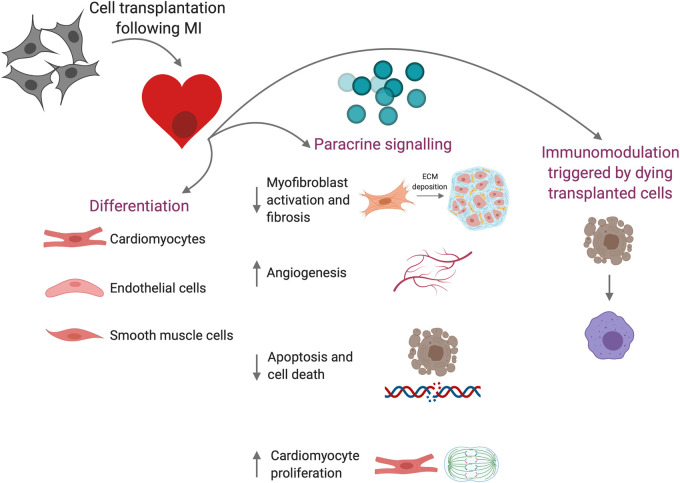
Proposed mechanisms of action of cardiac cell therapy. Cell transplantation following MI may contribute to cardiac repair via differentiation into cardiovascular cell types, the secretion of paracrine factors that modulate the host tissue's response to injury, or via immunomodulation triggered by the death of the transplanted cells. Created with BioRender.com.

Mechanisms responsible for the observed beneficial effects following administration of cardiac stromal cells or CPCs in both animal models and clinical trials have therefore been under debate (Figure 2). It has been suggested that the production of paracrine factors, such as growth factors, cytokines, and microRNAs [107], may stimulate endogenous regeneration or alter the tissue's response to injury. MicroRNAs are emerging players in cardiomyocyte proliferation and have been shown to modulate it by predominantly targeting components of the Hippo pathway [108]. For example, microRNA-199a, which promotes cardiomyocyte proliferation by targeting the Hippo pathway regulators TAOK1 and β-TrCP, was found to be expressed in CDC-derived extracellular vesicles [60,108,109]. Delivery of the secretome of MSCs or CPCs has been shown to be sufficient to induce cardiac repair following MI [60,110,111]. For example, administration of exosomes isolated from human CDCs significantly repressed scarring and improved cardiac function in a porcine MI model [111]. Furthermore, the therapeutic benefit of cardiac cell therapy may be mediated through stimulation of an acute immune response [112]. Injection of bone marrow mononuclear cells, either viable or freeze/thaw-killed, induced CCR2^+^ and CX3CR1^+^ macrophages and enhanced cardiac function in a mouse MI model.

If CPCs are to act as a ‘paracrine factors factory’ following transplantation or to stimulate an immune response, the problem of the extremely low cell retention must be overcome. Following MI, harsh events such as ischaemia, oxidative stress and increased inflammation limit the survival and engraftment of transplanted cells and typically 90% of the cells are lost within the first week [113–116]. Enhancing the survival of the cells may enable the window of beneficial paracrine signalling to be prolonged. Hence, the move towards using improved delivery strategies including biomaterials [117], simultaneous delivery of growth factors [118], and preconditioning techniques such as cell glycoengineering [119], and hypoxic conditioning [120,121] to enhance cell retention.

## Clinical trials

Since the early days of clinical studies into cardiac cell therapy, trials have produced conflicting results which emphasised the complexity of this approach. In 2005, the REPAIR-AMI trial reported an improvement in cardiac function following infusion of bone marrow cells post MI, whereas the ASTAMI study showed no benefit after transplantation of a comparable cell population in a similar patient cohort [122].The differences in the data may result from handling of the cell population *in vitro* or the method of analysing cardiac function [123]. In 2018, a review of meta-analyses of clinical trials using bone marrow cells concluded that the potential beneficial effect of cell therapy for the heart was still inconclusive despite over 20 years of study [124]. They reported that most clinical trials were statistically underpowered, and the meta-analyses was confounded by inconsistencies between studies.

Similarly, although the safety of both autologous (e.g. CADUCEUS and CONCERT-HF) and allogenic (e.g. ALLSTAR and CAREMI) CPC transplantation has been shown, beneficial effects have been limited. The largest CPC trials were ALLSTAR and CONCERT-HF with 134 and 90 patients, respectively [69,90]. Tyler et al. highlighted the lack of adequate numbers of participants in cardiac cell therapy trials by referencing the GUSTO trial which enrolled 41 201 MI patients to show an absolute mortality difference of 1.1% between accelerated tissue plasminogen activator and streptokinase [125,126].

## Current state of play

Various types of CPCs or cardiac stromal populations have been investigated and shown to be effective in improving cardiac function in animal MI models despite a lack of long-term retention and ability to generate *de novo* cardiomyocytes. Nevertheless, CPCs entered clinical trials which demonstrated safety, but limited beneficial effect, and phase III data are lacking. More recent evidence has shown that most adult mammalian cardiomyocyte renewal is achieved via cardiomyocyte proliferation rather than a progenitor cell type [4,74,104–106] and the therapeutic effect of CPC populations has been attributed to paracrine signalling that can have anti-apoptotic, pro-angiogenic, anti-inflammatory and anti-fibrotic roles.

In cell therapy, the optimal cell population would be a safe and robust population that can exert a therapeutic effect and be isolated and expanded in a time- and cost-effective manner. Cardiac stromal populations have proven to be safe as no adverse effects directly linked to them have been reported in clinical trials of cardiac disease. Although PSC-derived cells have also provided a promising source, their use comes with the added cost of *in vitro* differentiation into a cardiac phenotype, increased risk of arrythmias, purification to eliminate undifferentiated cells that increase the risk of tumourigenicity, as well as the need for long-term immunosuppression in the case of embryonic stem cells [127–129]. Moreover, limited cell retention [129–132] suggests that the therapeutic benefit of PSC-derived cells may also be attributed to paracrine mechanisms [130,132–134]. To address suboptimal retention and maturation of PSC-derived cardiomyocytes, co-transplantation with stromal cells such as epicardial cells [96] and MSCs [135], metabolic reprogramming [136,137], and biomaterials such as engineered heart tissue patches [138] have been utilised. However, these patches also come with limitations including the lack of electromechanical coupling and insufficient vascularisation [138]. Clinical trials evaluating the safety of injecting allogenic IPS-derived cardiomyocytes are currently underway in Japan (NCT04696328), China (NCT03763136) and Germany (NCT04396899).

## Conclusions

It is now established that CPCs do not meet the agreed criteria of a true progenitor or stem cell. However, CPC research has been fundamental in further characterising the heterogeneity of the stromal and fibroblast compartments of the heart. Moreover, identifying the mechanisms responsible for regeneration in regenerative animal models and repair after cell transplantation opens prospects for finding alternative ways to induce these mechanisms. CPC populations may potentially be used in combinatorial therapy approaches to aid host tissue revascularisation or enhance the retention and integration of PSC-derived cardiomyocyte grafts. The therapeutic approach will, after all, depend on the patient's clinical presentation as there is no single ideal approach for all MI patients.

## Perspectives

- Unlike the neonatal mouse and zebrafish hearts, the adult mammalian heart cannot regenerate after myocardial infarction. Despite early promise from rodent studies, administration of progenitor cells has not translated to the clinic.
- Multiple cardiac progenitor cell populations have been proposed, with overlap in their morphology and gene expression. Retention after transplantation is low and therapeutic benefit is thought to result from immunomodulation or release of paracrine factors which stimulate angiogenesis and/or cardiomyocyte proliferation.
- For cells to act as therapeutic ‘paracrine factories’, it is important to increase donor cell retention and optimise release of key factors to stimulate repair.

## Open Access Statement

Open access for this article was enabled by the participation of University of Oxford in an all-inclusive *Read & Publish* pilot with Portland Press and the Biochemical Society under a transformative agreement with JISC.

## Abbreviations

CPCs: cardiac progenitor cells
EPDCs: epicardium-derived cells
ISL1: islet1
ISO: isoproterenol
MI: myocardial infarction
MSCs: mesenchymal stem cells
SCA1: stem cell antigen 1
SP: side population
WT1: Wilms tumour 1
